# Supplementary material for: Discovery of actinomycin L, a new member of the actinomycin family of antibiotics
Source: Sci Rep. 2022 Feb 18;12:2813. doi: 10.1038/s41598-022-06736-0 (PMC8857259; doi:10.1038/s41598-022-06736-0)
Supplement: Supplementary file 1 — Supplementary Information. [file 41598_2022_6736_MOESM1_ESM.pdf]

SUPPLEMENTAL INFORMATION

accompanying the manuscript

Discovery of actinomycin L, a new member of the actinomycin family of antibiotics

Nataliia Machushynets [a], Somayah S. Elsayed [a], Chao Du [a], Maxime A. Siegler [b], Mercedes de la Cruz [c], Olga Genilloud [c], Thomas Hankemeier [d] and Gilles P. van Wezel [a, #]

[a] *Molecular Biotechnology, Institute of Biology, Leiden University, Sylviusweg 72, 2333 BE, The Netherlands*

[b] *Department of Chemistry, The Johns Hopkins University, 3400 North Charles Street, Baltimore, Maryland 21218, United States*

[c] *Fundación MEDINA, Health Sciences Technology Park, Avda Conocimiento 34, 18016 Granada, Spain*

[d] *Leiden Academic Centre for Drug Research (LACDR), Leiden University, Einsteinweg 55, 2333 CC, Leiden, The Netherlands*

[#] Author for correspondence. Tel: +31 71 5274310; email: g.wezel@biology.leidenuniv.nl

SUPPLEMENTAL METHODS

Single Crystal X-ray Crystallography

All reflection intensities were measured at 100(2) K using a SuperNova diffractometer (equipped with Atlas detector) with Cu *K*α radiation (λ = 1.54178 Å) under the program CrysAlisPro (Version CrysAlisPro 1.171.39.29c, Rigaku OD, 2017). The same program was used to refine the cell dimensions and for data reduction. The structure was solved and refined with SHELXL-2018/3 (4). Analytical numeric absorption correction using a multifaceted crystal model was applied using CrysAlisPro. The temperature of the data collection was controlled using the system Cryojet (manufactured by Oxford Instruments). Prior to mounting the chosen single crystal on the diffractometer, crystals were placed in some Parabar 10312 on a microscope slide and cooled via a cold N_2(*g*) stream in order to protect the crystal from potential solvent loss. The H atoms were placed at calculated positions (unless otherwise specified) using the instructions AFIX 13, AFIX 23, AFIX 43, AFIX 137 or AFIX 147 with isotropic displacement parameters having values 1.2 or 1.5 U_{eq} of the attached C or O atoms. The H atoms attached to N2, N1', N2', N6', N7', N1" and N2" were found from difference Fourier maps, and their coordinates were refined pseudofreely using the DFIX instruction in order to keep the N–H bonds within an acceptable range. The structure is partly disordered.

The asymmetric unit contains one molecule of the target compound as well as some significant amount of lattice MeOH solvent molecules. Six solvent molecules were modelled as ordered with four being fully occupied and two with partial occupancy factors of 0.918(11) and 0.487(10). The contribution of the remaining amount of disordered lattice solvent molecules has been removed using the SQUEEZE procedure in Platon (5). Furthermore, the atoms C9", C10" and C11" are disordered over two orientations, and the occupancy factor of the major component of the disorder refines to 0.533(10). The absolute configuration has been established by anomalous-dispersion effects in diffraction measurements on the crystal, and the Flack and Hooft parameters refine to 0.08(5) and 0.06(5), respectively. The model has chirality S, S, R, R, R, S, S, S, R, R at C2', C2", C4', C4", C10'. C12', C18', C18", C23', C23", respectively. Structure visualization and image preparation was done using Mercury (2).

SUPPLEMENTAL TABLES AND FIGURES

Table S1. Accurate mass of $[M+H]^+$ and product ions of the PPLs analyzed be ESI-QTOF MS/MS.

Compound	Formula	Proposed ion	Measurement (*m/z*)	Calculated (*m/z*)	Diff (ppm)
**PPL0**	$C_{31}H_{46}N_5O_9$	$[M+H]^+$	632.3170	632.3295	-18.99
	$C_{31}H_{44}N_5O_8$	$[M+H-H_2O]^+$	614.3177	614.3189	-1.2
	$C_{25}H_{33}N_4O_7$	[M+H-(H-MeVal-OH)]$^+$	501.2347	501.2349	0.65
	$C_{22}H_{28}N_3O_6$	[M+H-(H-Sar-MeVal-OH)]$^+$	430.1969	430.1978	-0.84
	$C_{19}H_{35}N_4O_6$	[(H-Val-HyPro-Sar-MeVal-OH)+H]$^+$	415.2552	415.2556	0.214
	$C_{19}H_{33}N_4O_5$	[(H-Val-HyPro-Sar-MeVal-OH)+H-H_2O]$^+$	397.2441	397.2450	-1.124
	$C_{14}H_{26}N_3O_5$	[(H-Hyp-Sar-MeVal-OH)+H]$^+$	316.1870	316.1872	0.957
	$C_{14}H_{24}N_3O_4$	[(H-Hyp-Sar-MeVal-OH)+H-H_2O]$^+$	298.1761	298.1766	-0.110
	$C_{13}H_{22}N_3O_4$	[(H-Val-HyPro-Sar)+H]$^+$	284.1606	284.1610	0.413
	$C_{12}H_{12}NO_3$	[M+H-(H-Val-HyPro-Sar-MeVal-OH)]$^+$	218.0816	218.0817	1.973
	$C_9H_{19}N_2O_3$	[(H-Sar-MeVal-OH)+H]$^+$	203.1391	203.1395	0.399
	$C_6H_{14}NO_2$	[(H-MeVal-OH)+H]$^+$	132.1015	132.1024	-3.067
	$C_4H_6NO_2$	[Thr+H]$^+$	100.0389	100.0398	-4.048
**PPL1**	$C_{31}H_{46}N_5O_8$	$[M+H]^+$	616.3326	616.3346	-2.417
	$C_{31}H_{44}N_5O_7$	$[M+H-H_2O]^+$	598.3248	598.3241	2.131
	$C_{25}H_{33}N_4O_6$	[M+H-(H-MeVal-OH)]$^+$	485.2392	485.2400	-0.538
	$C_{22}H_{28}N_3O_5$	[M+H-(H-Sar-MeVal-OH)]$^+$	414.2025	414.2028	0.368
	$C_{19}H_{35}N_4O_5$	[(H-Val-Pro-Sar-MeVal-OH)+H]$^+$	399.2607	399.2607	1.261
	$C_{19}H_{33}N_4O_4$	[(H-Val-Pro-Sar-MeVal-OH)+H-H_2O]$^+$	381.2504	381.2501	2.014
	$C_{17}H_{21}N_2O_4$	[M+H-(H-Pro-Sar-MeVal-OH)]$^+$	317.1496	317.1501	0.052
	$C_{14}H_{26}N_3O_4$	[(H-Pro-Sar-MeVal-OH)+H]$^+$	300.1916	300.1923	-0.609
	$C_{14}H_{24}N_3O_3$	[(H-Pro-Sar-MeVal-OH)+H-H_2O]$^+$	282.1809	282.1817	-1.127
	$C_{13}H_{22}N_3O_3$	[(H-Val-Pro-Sar)+H]$^+$	268.1657	268.1661	0.492
	$C_9H_{19}N_2O_3$	[(H-Sar-MeVal-OH)+H]$^+$	203.1392	203.1395	0.891
	$C_8H_{13}N_2O_2$	[(H-Pro-Sar)+H]$^+$	169.0968	169.0977	-2.095
	$C_6H_{14}NO_2$	[(H-MeVal-OH)+H]$^+$	132.1016	132.1024	-2.310
	$C_4H_6NO_2$	[Thr+H]$^+$	100.0391	100.0398	-2.049
**PPL2**	$C_{31}H_{44}N_5O_9$	$[M+H]^+$	630.3132	630.3139	-0.245
	$C_{31}H_{42}N_5O_8$	$[M+H-H_2O]^+$	612.3027	612.3033	-0.146
	$C_{25}H_{33}N_4O_8$	[M+H-(H-MeVal)]$^+$	517.2286	517.2298	-1.335
	$C_{25}H_{31}N_4O_7$	[M+H-(H-MeVal-OH)]$^+$	499.2190	499.2192	0.549
	$C_{22}H_{26}N_3O_6$	[M+H-(H-Sar-MeVal-OH)]$^+$	428.1814	428.1821	-0.495
	$C_{19}H_{33}N_4O_6$	[(H-Val-OxoPro-Sar-MeVal-OH)+H]$^+$	413.2395	413.2400	0.094
	$C_{19}H_{31}N_4O_5$	[(H-Val-OxoPro-Sar-MeVal-OH)+H-H_2O]$^+$	395.2293	395.2294	1.021
	$C_{14}H_{24}N_3O_5$	[(H-OxoPro-Sar-MeVal-OH)+H]$^+$	314.1711	314.1715	0.168
	$C_{14}H_{22}N_3O_4$	[(H-Val-OxoPro-Sar-MeVal-OH)+H-H_2O]$^+$	296.1605	296.1610	0.059
	$C_{13}H_{20}N_3O_4$	[(H-Val-OxoPro-Sar)+H]$^+$	282.1448	282.1453	-0.116
	$C_{12}H_{12}NO_3$	[M+H-(H-Val-OxoPro-Sar-MeVal-OH)]$^+$	218.0815	218.0817	1.514
	$C_9H_{19}N_2O_3$	[(H-Sar-MeVal-OH)+H]$^+$	203.1391	203.1395	0.399
	$C_6H_{14}NO_2$	[(H-MeVal-OH)+H]$^+$	132.1017	132.1024	-1.554
	$C_4H_6NO_2$	[Thr+H]$^+$	100.0391	100.0398	-2.049
**PPL3**	$C_{38}H_{50}N_7O_9$	$[M+H]^+$	748.3663	748.3670	-0.204
	$C_{38}H_{48}N_7O_8$	$[M+H-H_2O]^+$	730.3561	730.3564	0.290
	$C_{32}H_{37}N_6O_7$	[M+H-(H-MeVal-OH)]$^+$	617.2716	617.2723	-0.363
	$C_{26}H_{39}N_6O_6$	[(H-Val-AntPro-Sar-MeVal-OH)+H]$^+$	531.2928	531.2931	0.453
	$C_{26}H_{37}N_6O_5$	[(H-Val-AntPro-Sar-MeVal-OH)+H-H_2O]$^+$	513.2820	513.2825	0.010
	$C_{21}H_{30}N_5O_5$	[(H-AntPro-Sar-MeVal-OH)+H]$^+$	432.2247	432.2246	1.283
	$C_{21}H_{28}N_5O_4$	[(H-AntPro-Sar-MeVal-OH)+H-H_2O]$^+$	414.2137	414.2141	0.288
	$C_{20}H_{26}N_5O_4$	[(H-Val-AntPro-Sar)+H]+	400.1982	400.1984	0.673
	$C_{17}H_{21}N_4O_3$	[(H-Val-AntPro)+H]+	329.1607	329.1613	-0.36
	$C_{17}H_{21}N_2O_4$	[M+H-(H-AntPro-Sar-MeVal-OH)]$^+$	317.1493	317.1501	-0.894
	$C_{15}H_{17}N_4O_3$	[(H-AntPro-Sar)+H]$^+$	301.1295	301.1300	-0.056
	$C_9H_{19}N_2O_3$	[(H-Sar-MeVal-OH)+H]$^+$	203.1390	203.1395	-0.093
	$C_6H_{14}NO_2$	[(H-MeVal-OH)+H]$^+$	132.1014	132.1024	-3.824
	$C_4H_6NO_2$	[Thr+H]$^+$	100.0401	100.0398	7.947

Table S2. Gene organization of the actinomycins biosynthetic gene cluster in *Streptomyces* sp. MBT27 and similarities to corresponding protein sequences encoded by orthologues in the *S. antibioticus IMRU 3720* biosynthetic gene cluster.

***S. antibioticus IMRU 3720* gene**	**Function**	***Streptomyces* sp. MBT27 actinomycin cluster ORF**	**Identity**	**Similarity**
saacmT	Hypothetical protein	ORF01	0.80	0.89
saacmS	Hypothetical protein	ORF02	0.84	0.93
saacmR	MbtH-like protein	ORF03	0.86	0.94
saacmD	4-MHA carrier protein AcmACP	ORF04	0.60	0.69
saacmA	Peptide synthetase ACMS I	ORF05	0.71	0.78
saacmB	Peptide synthetase ACMS II	ORF06	0.70	0.78
saacmC	Peptide synthetase ACMS III	ORF07	0.77	0.85
saacmE	Hypothetical protein	ORF08	0.91	0.93
saacmF	Aryl formamidase	ORF09	0.79	0.82
saacmG	Tryptophan 2,3-dioxygenase	ORF10	0.83	0.89
saacmK	Kynureninase	ORF11	0.86	0.91
saacmL	Methyltransferase	ORF12	0.88	0.93
saacmM	Cytochrome P450	ORF13	0.85	0.88
saacmN	Ferredoxin	ORF14	0.69	0.78
saacmO	LmbU-like protein	ORF15	0.72	0.80
saacmP	TetR family transcriptional regulator	ORF16	0.77	0.86
saacmQ	Siderophore-interacting protein	ORF17	0.76	0.85
saacmrA	ABC transporter ATPase subunit	ORF18	0.89	0.93
saacmrB	ABC 2-type transporter	ORF19	0.93	0.96
saacmrC	UvrA-like protein	ORF20	0.86	0.90

Table S3. X-ray crystallography data.

	xs2313a
Crystal data	
Chemical formula	$C_{69}H_{90}N_{14}O_{17}·5.405(CH_4O)$
M_r	1560.87
Crystal system, space group	Trigonal, $P3_221$
Temperature (K)	100
a, *c* (Å)	18.4731 (2), 42.4216 (5)
V (Å^3)	12537.1 (3)
Z	6
Radiation type	Cu $K\alpha$
μ (mm^{-1})	0.77
Crystal size (mm)	0.23 × 0.15 × 0.07
Data collection	
Diffractometer	SuperNova, Dual, Cu at zero, Atlas
Absorption correction	Analytical *CrysAlis PRO* 1.171.40.53 (Rigaku Oxford Diffraction, 2019) Analytical numeric absorption correction using a multifaceted crystal model based on expressions derived by (1). Empirical absorption correction using spherical harmonics, implemented in SCALE3 ABSPACK scaling algorithm.
T_{min}, T_{max}	0.897, 0.957
No. of measured, independent and observed [$I > 2\sigma(I)$] reflections	90598, 14994, 13477
R_{int}	0.043
(sin θ/λ)$_{max}$ (Å^{-1})	0.598
Refinement	
$R[F^2 > 2\sigma(F^2)]$, $wR(F^2)$, S	0.046, 0.128, 1.03
No. of reflections	14994
No. of parameters	1091
No. of restraints	79

H-atom treatment	H atoms treated by a mixture of independent and constrained refinement
$\Delta\rho_{max}$, $\Delta\rho_{min}$ (e Å^{-3})	0.68, -0.26
Absolute structure	Flack x determined using 5493 quotients [(I+)-(I-)]/[(I+)+(I-)] (3).
Absolute structure parameter	0.08 (5)

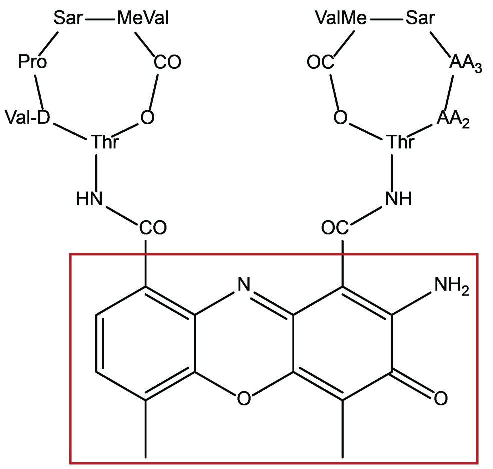

Fig. S1 Chemical structure of actinomycin. Actinomycins are composed of a chromophore group highlighed with the red square and two pentapeptide chains, where aminoacid AA2 and AA3 differs depending on the actinomycin group.

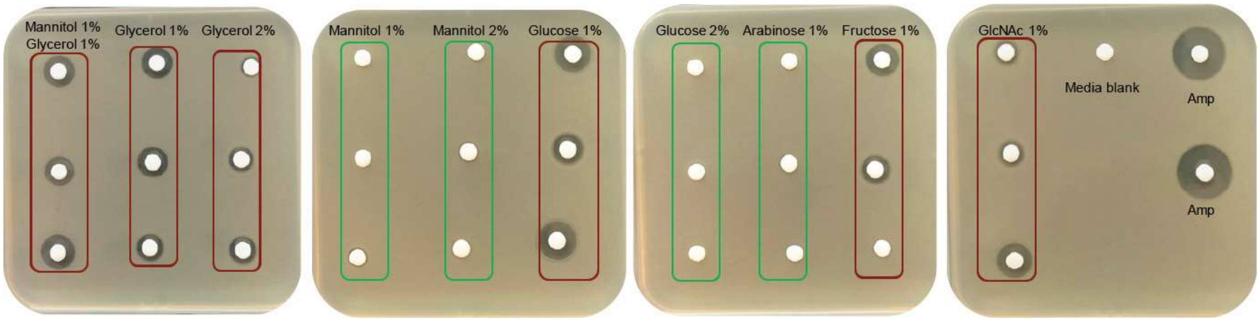

Fig. S2 Representative results of antimicrobial activity of *Streptomyces* sp. MBT27 extracts against *B. subtilis.* *Streptomyces* sp. MBT27 was fermented in minimal medium (MM) with different carbon sources, namely (percentages in w/v): 1% of both mannitol and glycerol, 1% mannitol, 2% mannitol, 1% glycerol, 2% glycerol, 1% glucose, 2% glucose, 1% fructose, 1% arabinose, or 1% *N*-acetylglucosamine (GlcNAc), and extracted with ethyl acetate. The red boxes indicate active extracts of *Streptomyces* sp. MBT27, while the green boxes represent extracts without antibacterial activity against *B. subtilis*. Ampicillin was used as a positive control. Note, that extracts of cultures fermented with different carbon sources showed different bioactivity profiles.

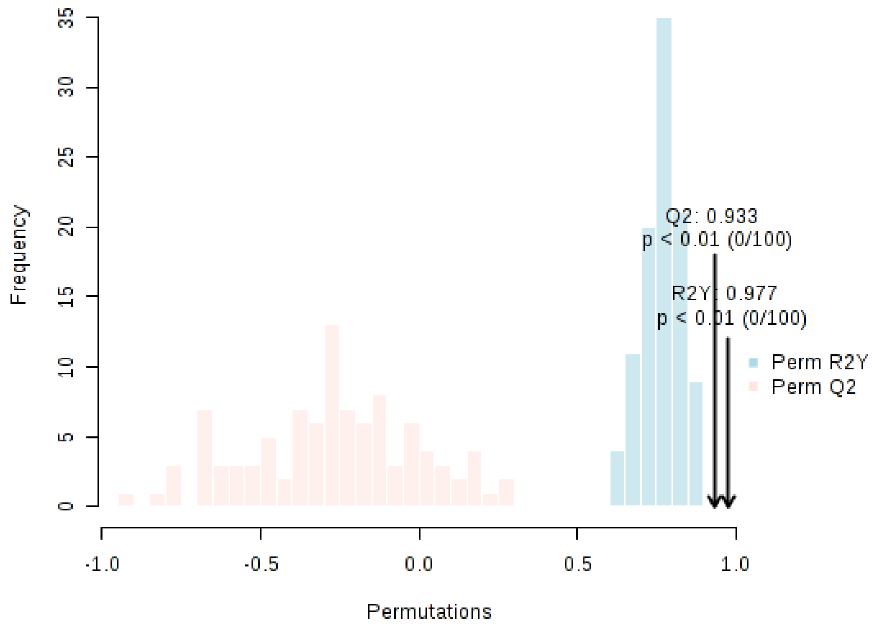

Fig. S3 Permutation validation of OPLS-DA model.

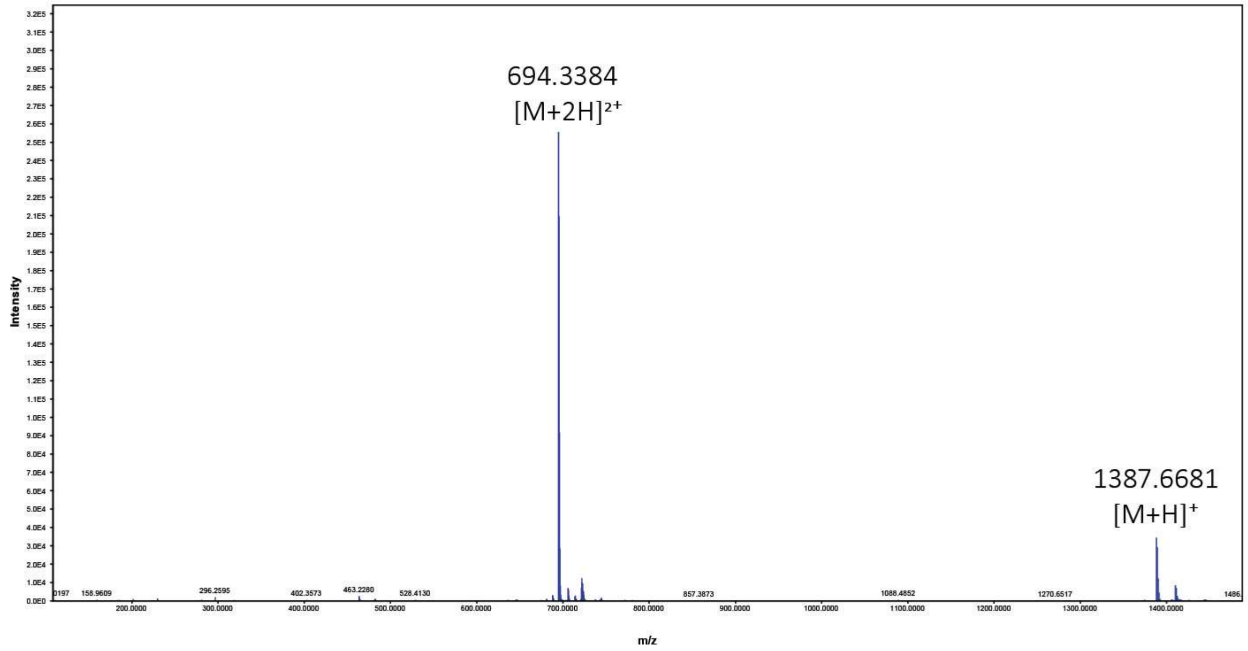

Fig. S4 HRMS spectrum of **1.**

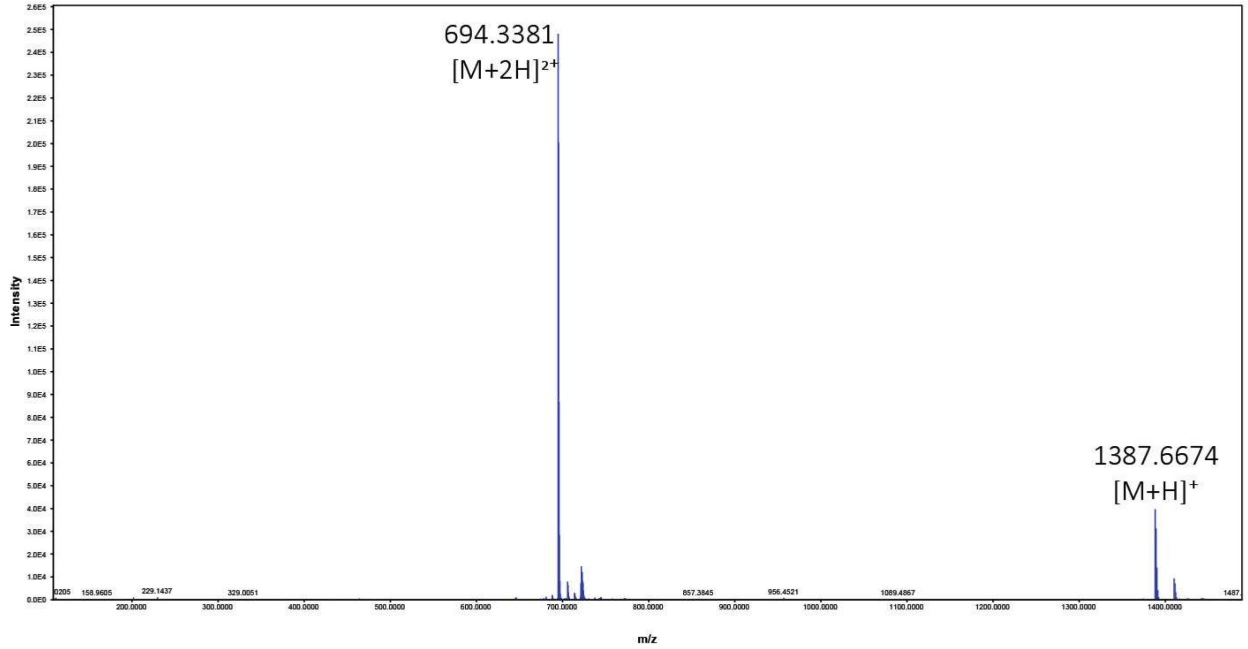

Fig. S5 HRMS spectrum of **2.**

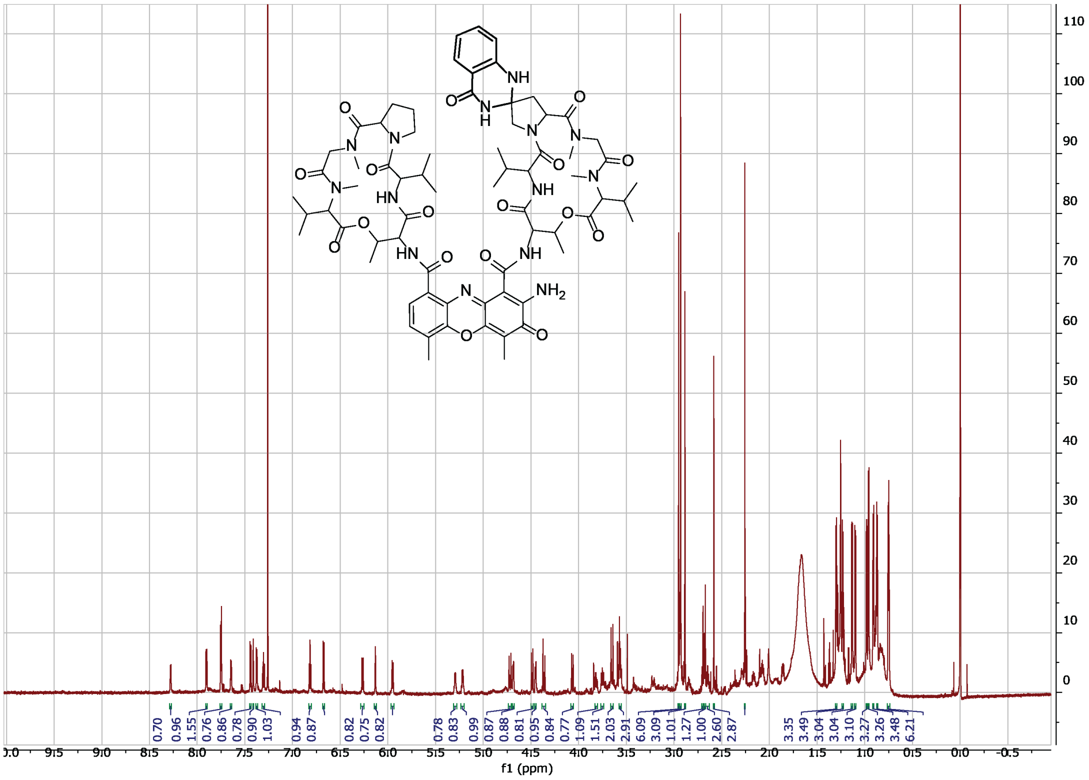

Fig. S6 ^{1}H NMR spectrum of **1** (850 MHz, in $CDCl_3$ with TMS).

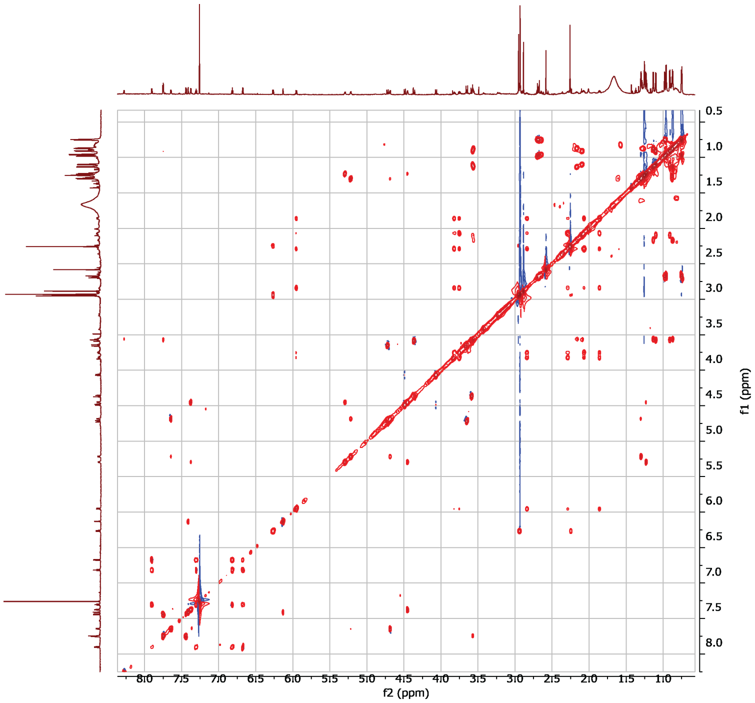

Fig. S7 ^{1}H–^{1}H TOCSY spectrum of **1** (850 MHz, in $CDCl_3$ with TMS).

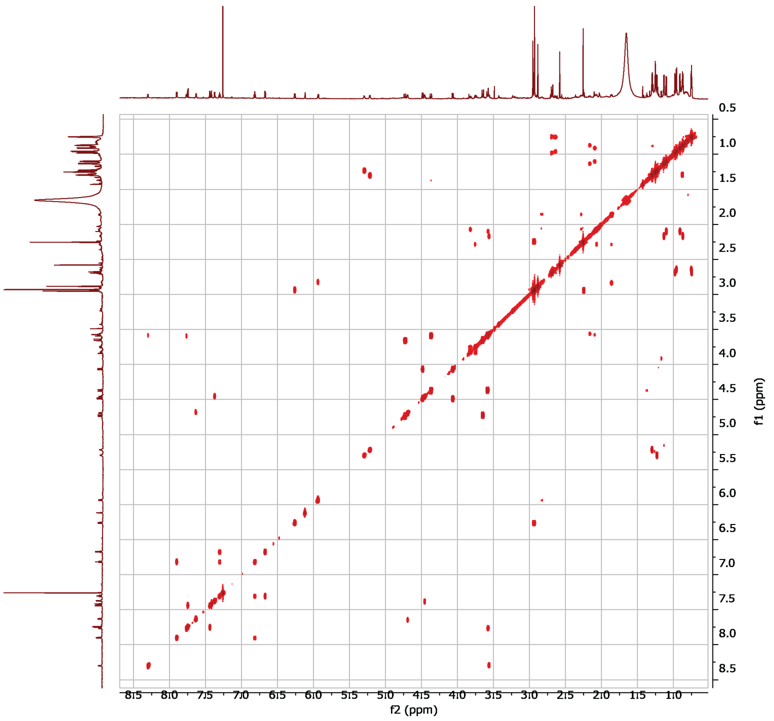

Fig. S8 ^1H–^1H COSY spectrum of **1** (850 MHz, in $CDCl_3$ with TMS).

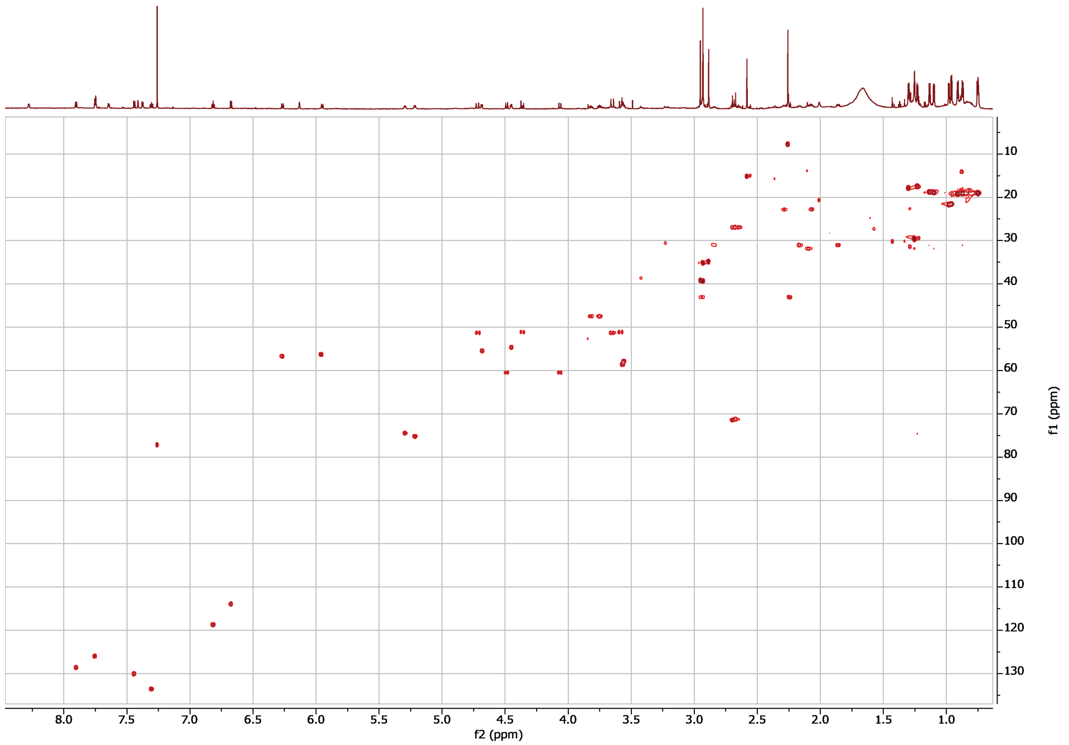

Fig. S9 HSQC spectrum of **1** (850 MHz, in $CDCl_3$ with TMS).

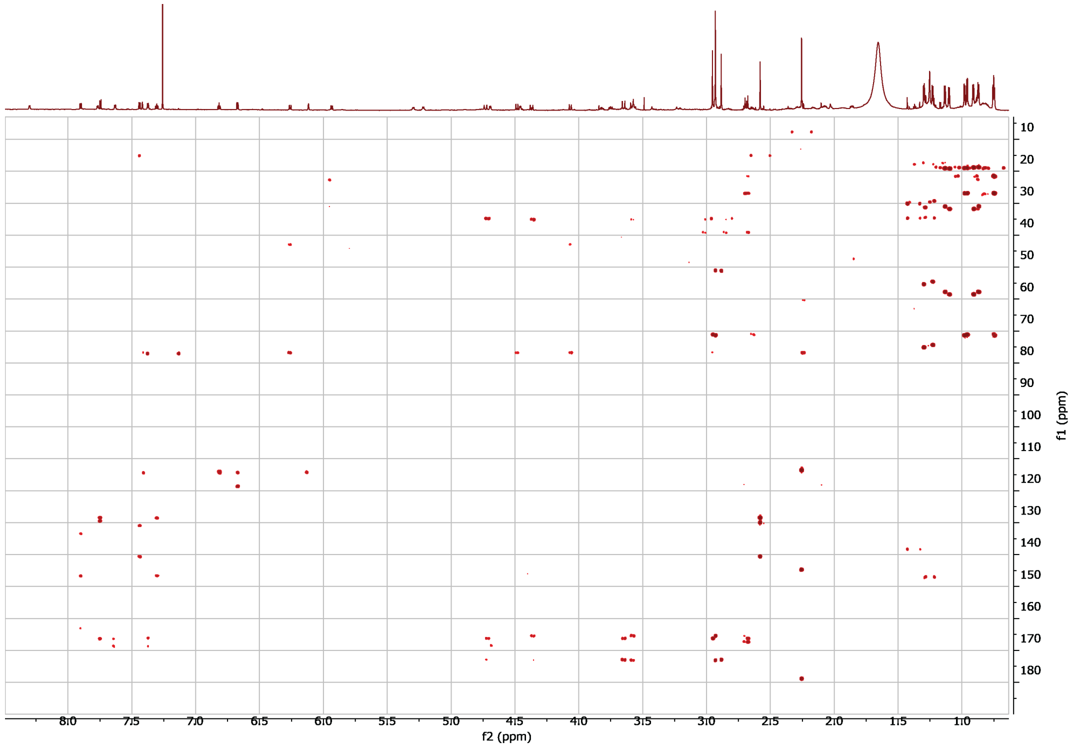

Fig. S10 HMBC spectrum of **1** (850 MHz, in $CDCl_3$ with TMS).

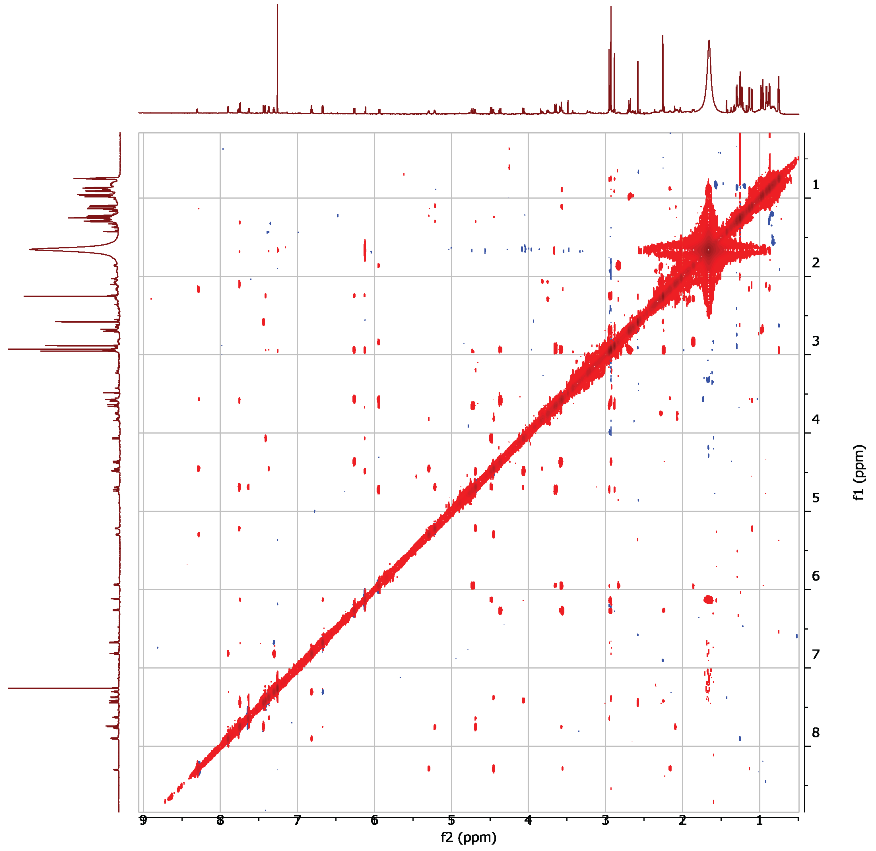

Fig. S11 NOESY spectrum of **1** (850 MHz, in $CDCl_3$ with TMS).

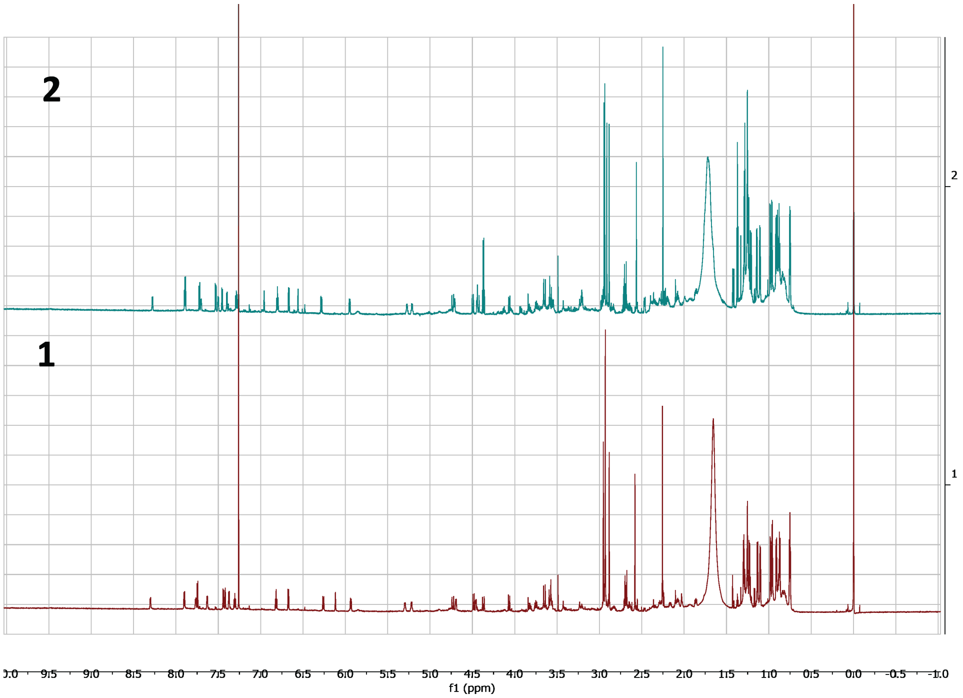

Fig. S12 Stacked ^{1}H NMR spectra of **1** and **2** (850 MHz, in $CDCl_3$ with TMS).

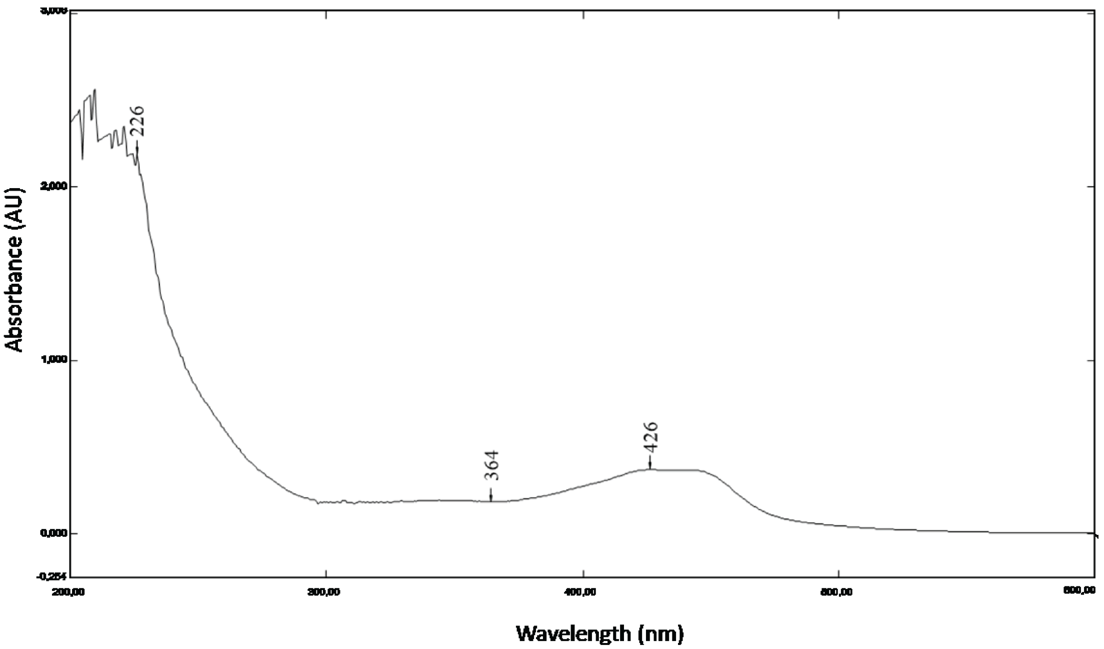

Fig. S13 UV spectrum of **1.**

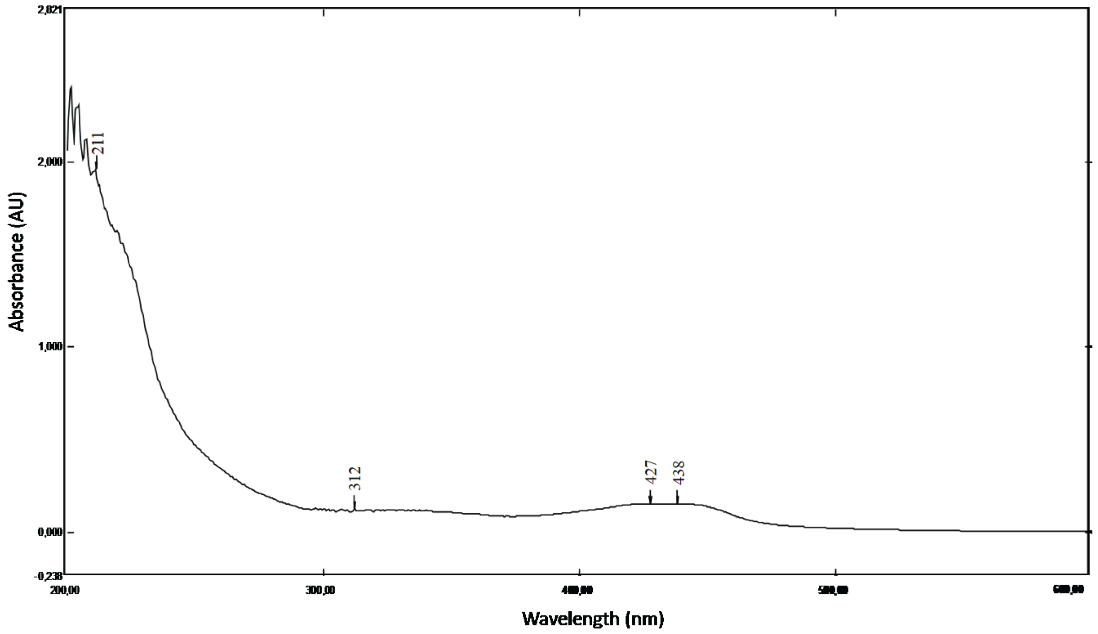

Fig. S14 UV spectrum of **2.**

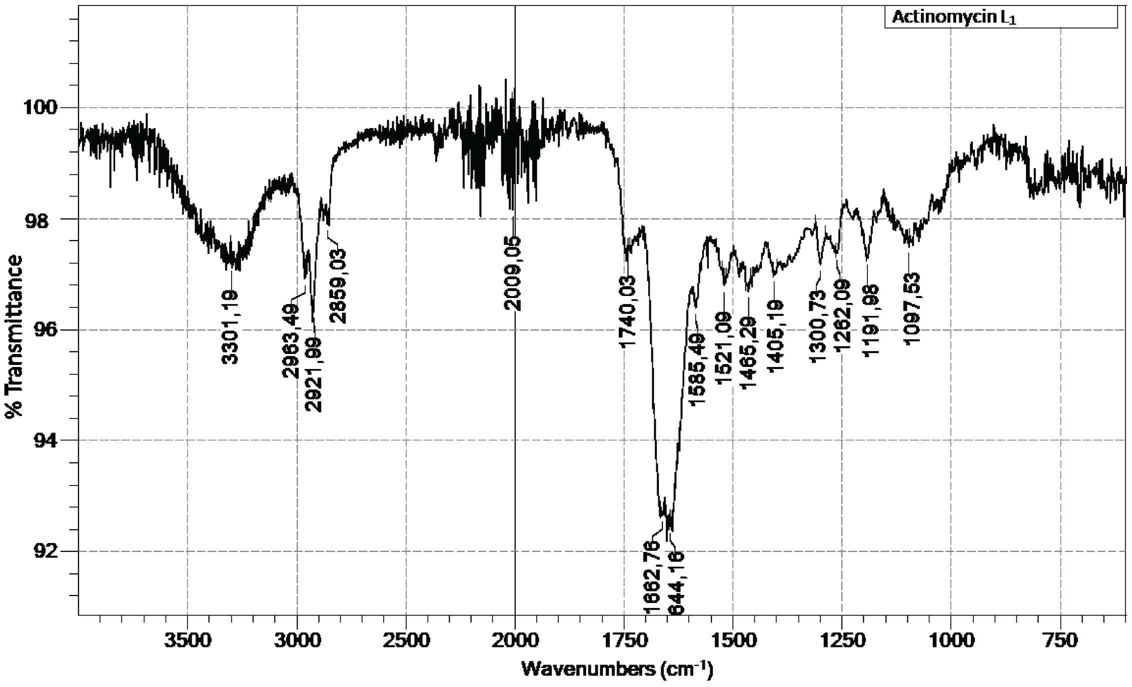

Fig. S15 IR spectrum of **1.**

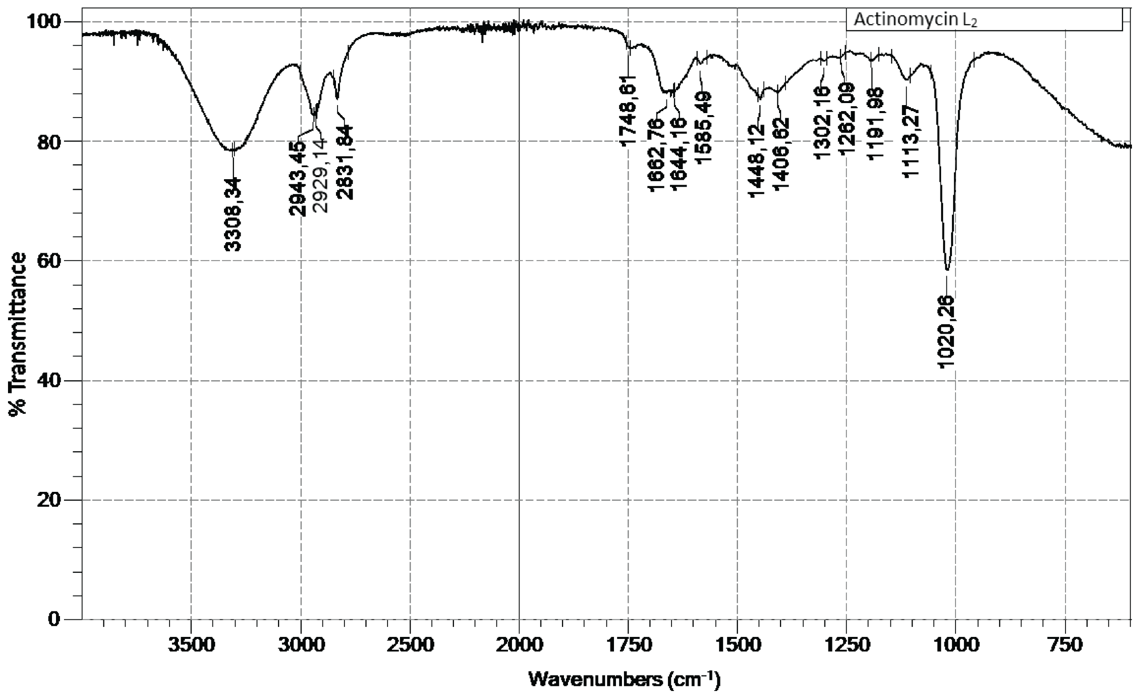

Fig. S16 IR spectrum of **2.**

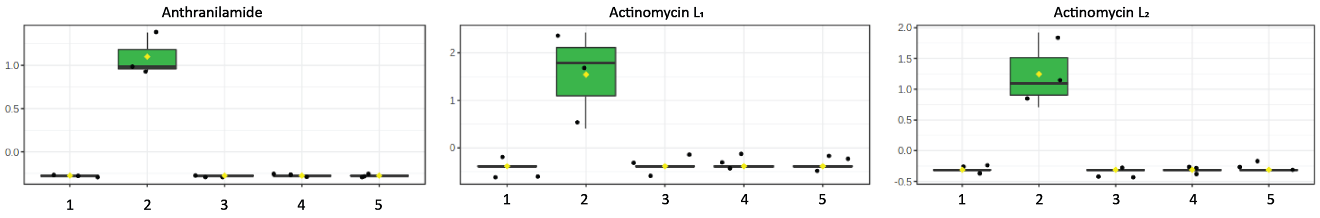

Fig. S17 *S. antibioticus* is incapable to produces actinomycin L unless anthranilamide is supplied. Box plots showing the relative intensities of anthranilamide, actinomycin L_1 and L_2 after log transformation and pareto scaling in the cultures of *S. antibioticus* fermented for seven days in MM with different carbon sources 1. 1% fructose; 2. 1% fructose + 0.7 mM anthranilamide; 3. 1% glycerol; 4. 1% mannitol + 1 % glycerol; 5. 2% glycerol (all %ages in w/v).

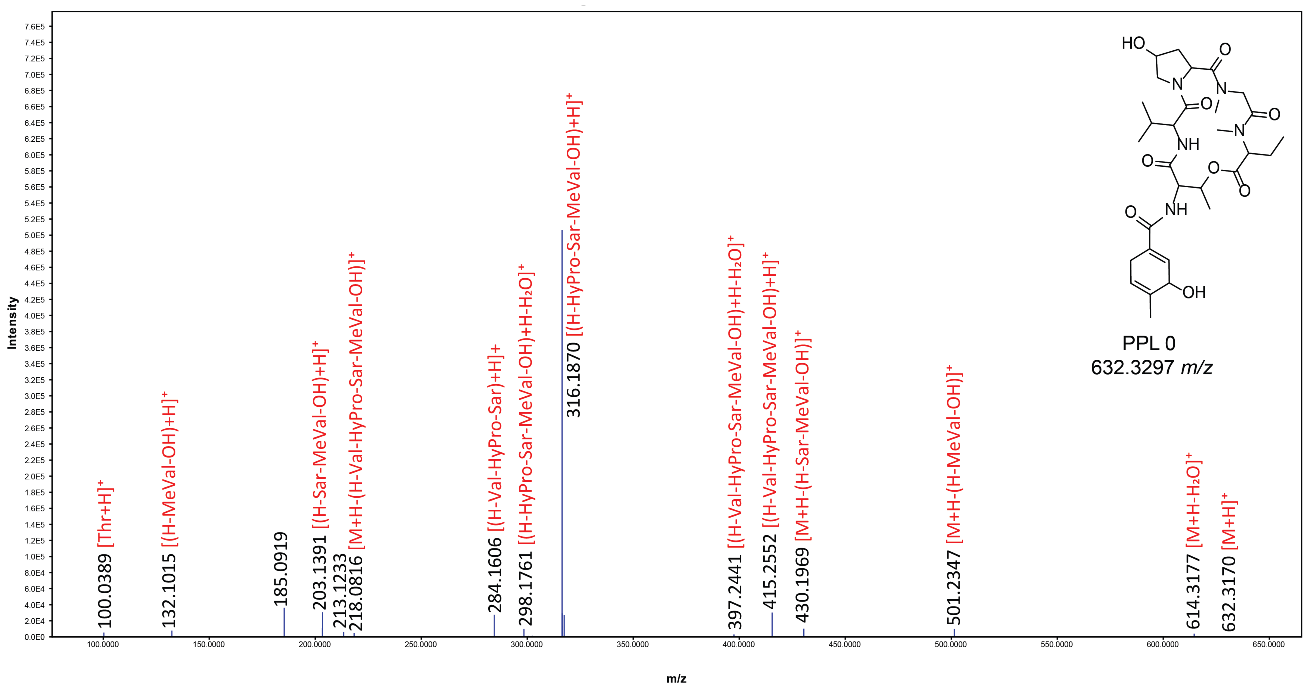

Fig. S18 QTOF MS/MS spectrum of 4-MHB-containing pentapeptide lactone PPL 0.

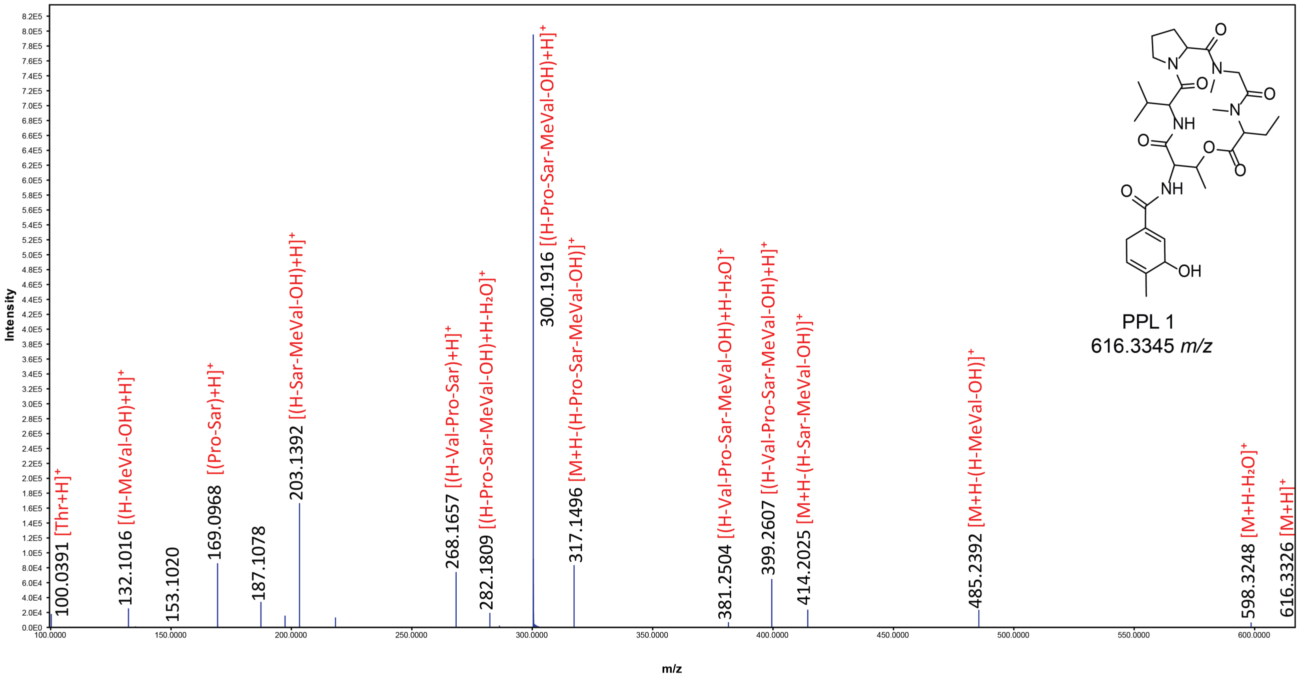

Fig. S19 QTOF MS/MS spectrum of 4-MHB-containing pentapeptide lactone PPL 1.

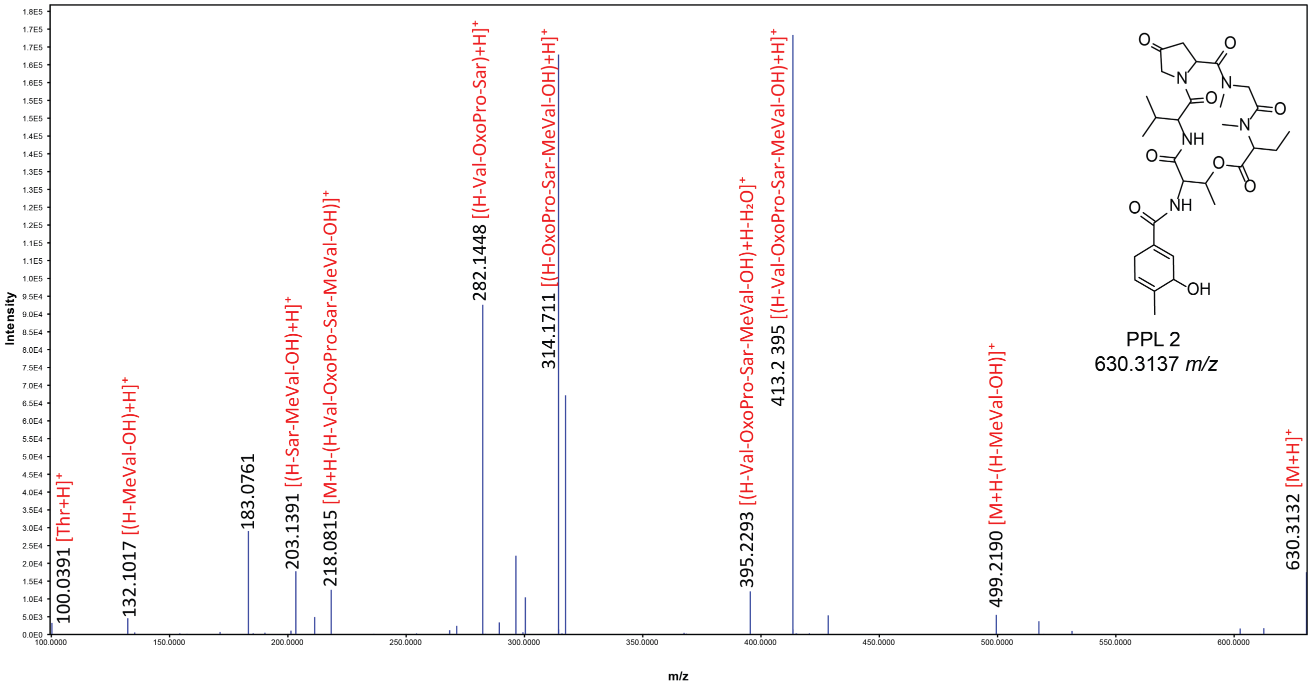

Fig. S20 QTOF MS/MS spectrum of 4-MHB-containing pentapeptide lactone PPL 2.

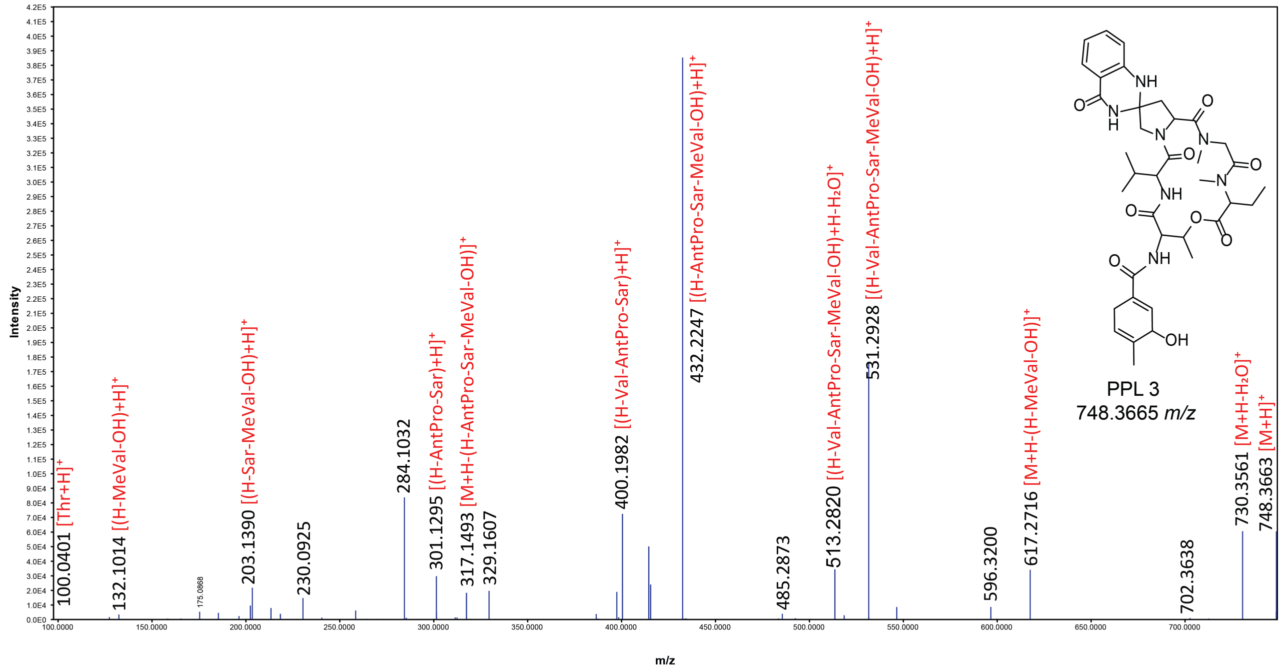

Fig. S21 QTOF MS/MS spectrum of 4-MHB-containing pentapeptide lactone PPL 3.

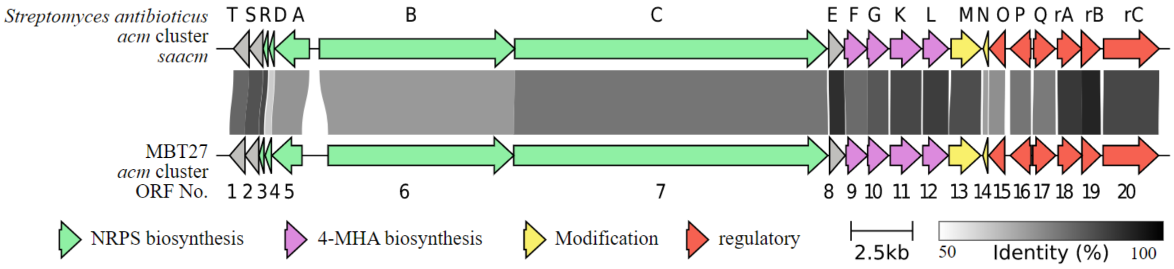

Fig. S22 Alignment of actinomycin biosynthetic gene cluster from *S. antibioticus* and *Streptomyces* sp. MBT27. Grey arrows indicate genes with unknown function. Grey bars connecting homologues pairs from the two cluster, the identity of two genes is indicated by different levels of greyness.

REFERENCES

1. Clark RC, and Reid JS. 1995. The analytical calculation of absorption in multifaceted crystals. Acta Crystallogr A 51:887-897.
2. Macrae CF, Sovago I, Cottrell SJ, Galek PTA, McCabe P, Pidcock E, Platings M, Shields GP, Stevens JS, Towler M and Wood PA. 2020. Mercury 4.0: from visualization to analysis, design and prediction J. Appl. Cryst 53, 226-235.
3. Parsons S, Flack HD, and Wagner T. 2013. Use of intensity quotients and differences in absolute structure refinement. Acta Crystallogr B 69:249-259.
4. Sheldrick G. 2015. SHELXT - Integrated space-group and crystal-structure determination. Acta Crystallogr A 71:3-8.
5. Spek A. 2009. Structure validation in chemical crystallography. Acta Crystallographica Section D 65:148-155.
